# Bifunctional EGFR/CD44-Targeted Diabody-Decorated Doxorubicin-Loaded Solid Lipid Nanoparticles for Targeted Therapy in Triple-Negative Breast Cancer

**DOI:** 10.34172/apb.45929

**Published:** 2026-05-31

**Authors:** Melika Arastehfar, Meysam Soleimani Badie, Katayoun Derakhshandeh

**Affiliations:** ^1^Department of Pharmaceutics, School of Pharmacy, Medicinal Plants and Natural Products Research Center, Hamedan University of Medical Sciences, Hamedan, Iran; ^2^Department of Pharmaceutical Biotechnology, School of Pharmacy, Hamadan University of Medical Sciences, Hamadan, Iran

**Keywords:** Diabody, Solid lipid nanoparticle, Doxorubicin, Breast cancer

## Abstract

**Purpose:**

Triple-negative breast cancer (TNBC) is an aggressive subtype of breast cancer that lacks targeted therapies, making its treatment highly challenging. This study aimed to design a novel dual-targeted drug delivery system to improve therapeutic efficacy and reduce the side effects of conventional chemotherapy.

**Methods:**

Solid lipid nanoparticles (SLNs) were synthesized using a double emulsion solvent evaporation method to encapsulate doxorubicin. These SLNs were then covalently conjugated with a diabody targeting both epidermal growth factor receptor (EGFR) and CD44, which are overexpressed in TNBC cells. The resulting nanoparticles were characterized in terms of size, morphology, zeta potential, and encapsulation efficiency. In vitro cytotoxicity was assessed using MDA-MB-468 TNBC cells.

**Results:**

The optimized dual-targeted SLNs exhibited appropriate size (~116 nm), high encapsulation efficiency (~79%), and good stability. In vitro assays demonstrated that diabody-conjugated SLNs significantly enhanced cellular uptake and cytotoxicity compared to both free doxorubicin and untargeted SLNs, particularly at higher concentrations.

**Conclusion:**

The results support the potential of diabody-conjugated SLNs as an effective and selective drug delivery system for TNBC. This approach offers improved therapeutic outcomes and reduced systemic toxicity, suggesting its promise for future clinical applications in targeted breast cancer therapy.

## Introduction

 Breast cancer is the most prevalent cancer among women worldwide.^1^ Among the various subtypes of breast cancer, Triple-Negative Breast Cancer (TNBC) is one of the most aggressive forms.^2,3^ Due to its lack of well-established diagnostic markers, TNBC is difficult to detect. Moreover, the heterogeneity of cancer presents significant challenges in targeted drug delivery.^4^ CD44 and EGFR proteins were selected due to their overexpression in TNBC cells. CD44 is expressed in approximately 80–90% and also EGFR in more than 50% of stem-like cell populations, enabling dual targeting for enhanced specificity and reduced off-target effects. Epidermal Growth Factor Receptor (EGFR) is overexpressed in TNBC cells^5^, while CD44 is an important marker on the surface of breast cancer stem cells, which is also highly expressed in TNBC cells compared to other types of breast cancer.^6^ Doxorubicin, an anthracycline drug, is one of the most widely used chemotherapy agents for cancer treatment.^7^ However, its use is often limited by serious side effects such as cardiotoxicity, alopecia, and gastrointestinal issues.^8^ These side effects often lead to dose-limiting toxicity and compromise the therapeutic efficacy of doxorubicin, necessitating the development of more efficient drug delivery systems.

 To mitigate these side effects and improve therapeutic efficacy, lipid-based nanoparticles (Solid Lipid Nanoparticles, SLNs) encapsulating doxorubicin have been developed.^9^ These SLNs can minimize drug exposure to non-target tissues while enhancing drug delivery to cancer cells.^10^ SLNs have demonstrated advantages such as controlled drug release, enhanced stability, and biocompatibility, making them an attractive option for chemotherapeutic drug delivery.^9,10^

 In this study, we aim to synthesize solid lipid nanoparticles (SLNs) conjugated with a dual-functional diabody targeting both CD44 and EGFR.^11^ The goal is to create a targeted delivery system for doxorubicin to specifically attack TNBC cells, thereby enhancing therapeutic efficacy and reducing side effects. The diabody, a genetically engineered antibody, plays a critical role in targeting the nanoparticles to the cancer cells, improving the specificity and uptake of the drug.^12^

 Previous studies have demonstrated that the combination of nanoparticles with targeting ligands, such as antibodies or diabodies, can significantly improve the selectivity of drug delivery systems for cancer cells, reducing off-target effects.^13,14^

 The research utilizes a double emulsion (w/o/w) method to prepare doxorubicin-loaded SLNs, followed by conjugation of the SLNs with the dual-functional diabody. The physicochemical properties of the nanoparticles, such as size, zeta potential, and drug encapsulation efficiency, will be analyzed. Additionally, the cytotoxicity of the doxorubicin-loaded SLNs, with and without the diabody conjugation, will be evaluated in TNBC cell lines using the reMTT assay. This study aims to overcome the challenges posed by conventional drug delivery systems, particularly in TNBC, where existing therapies often fail.

 Moreover, the combination of a dual-targeting diabody with SLNs may also improve the pharmacokinetic profile of doxorubicin by enhancing its stability and prolonging its circulation time in the bloodstream.

 The significance of this study lies in its potential to improve the targeted delivery of chemotherapy drugs, particularly in TNBC, where current treatment options are often ineffective. By combining the targeting capabilities of a diabody with the drug-delivery efficiency of SLNs, this research may pave the way for more effective and safer cancer therapies.

## Materials and Methods

###  Materials

 Doxorubicin hydrochloride was purchased from Beijing Zhongshuo Pharmaceutical Technology Development Co., Ltd. (Beijing, China). Stearic Acid, Soy Lecithin, Glycerol Monostearate(GMS), 1-Ethyl-3-(3-dimethylaminopropyl) carbodiimide (EDC), and N-Hydroxysuccinimide (NHS) form Sigma-Aldrich, USA. Tween 80, Chloroform (Merck, Germany).

 Nickel-Nitrilotriacetic acid (Ni-NTA) Resin (Sigma-Aldrich, USA)

 Phosphate-Buffered Saline (PBS), Tris-HCl buffer, Dimethyl sulfoxide (DMSO), Methanol, and Ethanol were prepared from Sigma-Aldrich, USA.

 All reagents were of analytical grade to ensure the highest purity and accuracy during synthesis and characterization.

## Methods

###  Preparation of Solid Lipid Nanoparticles (SLNs) Containing Doxorubicin

####  Preparation of Doxorubicin-loaded SLNs

 The SLNs were prepared using a double emulsion (w/o/w) method^15^, a widely used technique for the encapsulation of hydrophilic drugs like doxorubicin.

- Step 1: Doxorubicin was dissolved in a small amount (200 µL) of deionized water.
- This solution was then emulsified in an organic phase.
- Step 2: The first aqueous phase containing Doxorubicin was added dropwise into the lipid phase consisting of stearic acid, soy lecithin, and glycerol monostearate in chloroform (10 mL total), followed by homogenization at 11,000 rpm using a high-speed homogenizer (e.g., Silent Crusher M, Heidolph, Germany).
- Step 3: The mentioned obtained phase was added dropwise to a second aqueous phase containing 200 mg of Tween 80 dissolved in 20 mL of water. This process was conducted at 60°C and 11,000 rpm. The homogenization continued for an additional 5 minutes. Subsequently, the suspension was stirred in an ice bath for 10–15 minutes using a magnetic stirrer. Finally, it was stirred at room temperature for 2 hours to ensure complete evaporation of the organic solvent. Residual chloroform was assessed via gas chromatography (GC, Agilent 7890A) with flame ionization detection (FID), confirming levels < 0.01% post-evaporation (below ICH Q3C limits of 60 ppm).^16^
- Step 4: The remaining solution was lyophilized^17^ to obtain a dry powder of doxorubicin-loaded SLNs using a programmable freeze dryer (Shin PVTFD10R, Shinil Lab, Korea). Prior to the freeze-drying process, the sample was placed in a -80°C freezer for a minimum of an overnight to ensure complete freezing. ​The samples were transferred to the freeze-dryer, operated at -55°C, and lyophilized for 24 hours.

In this study, the lipid composition and the volume of the aqueous phase were varied to create different formulations of solid lipid nanoparticles (SLNs). The factors considered as independent variables were the concentration of stearic acid and glycerol monostearate as lipid compositions and the volume of the aqueous phase. The particle size and encapsulation efficiency (EE%) were the responses.

 In total, 15 different formulations were prepared based on the following factors, and the experimental settings were carried out three times for each formulation (Table 1).

**Table 1:** Formulation results, including particle size and encapsulation efficiency (EE%).

**Run**	**Stearic acid (mg)**	**GMS (mg)**	**Aqueous phase volume (ml)**	**Size (mean±SD)**	**EE% (mean±SD)**
1	50	50	5	295.98 ± 5.10	72.81 ± 1.45
2	50	50	10	247.37 ± 6.20	76.62 ± 1.32
3	50	50	15	268.18 ± 7.05	68.17 ± 1.10
4	50	50	20	116.2 ± 3.30	78.78 ± 0.75
5	50	50	25	204.34 ± 4.75	82.55 ± 0.90
6	75	75	5	217.99 ± 4.20	81.13 ± 1.05
7	75	75	10	187.91 ± 5.60	75.57 ± 1.25
8	75	75	15	292.65 ± 6.90	80.88 ± 0.95
9	75	75	20	145.84 ± 4.00	84.42 ± 1.10
10	75	75	25	225.62 ± 4.50	79.65 ± 1.15
11	100	100	5	151.96 ± 5.80	62.82 ± 1.35
12	100	100	10	345.18 ± 7.00	62.23 ± 1.50
13	100	100	15	171.35 ± 5.15	81.84 ± 0.85
14	100	100	20	225.66 ± 5.40	71.72 ± 1.00
15	100	100	25	276.61 ± 6.30	78.22 ± 1.20

####  Evaluation of Doxorubicin-Loaded SLNs

 The physicochemical properties of the prepared SLNs were characterized by the following methods

###  Particle Size and Distribution

 The average particle size and polydispersity index (PDI) were recorded to evaluate the uniformity of the particle size. The size distribution of the nanoparticles measurements (Malvern ZEN3600) used 1:10 dilution in water (viscosity 0.887 mPa·s, refractive index 1.33), 173° scattering angle, disposable cuvette, 3 runs at 25°C, with n = 3 replicates.^18^

###  Zeta Potential 

 The surface charge of the nanoparticles was measured to assess their suspension stability. A higher zeta potential indicates better stability of the nanoparticles due to electrostatic repulsion between particles.^19^

###  Entrapment Efficiency (EE)

 The entrapment efficiency of doxorubicin in the nanoparticles was calculated by determining the difference between the total drug concentration and the free drug concentration in the supernatant^13^. The drug concentration was measured using UV spectrophotometry at a wavelength of 480 nm.


$$EE\%=\frac{Total\;amount\;of\;drug-unloaded\;drug}{Total\;amount\;of\;drug}\times 100$$

###  Morphological Characterization

 The morphology of the doxorubicin-loaded solid lipid nanoparticles (SLNs) was analyzed using Scanning Electron Microscopy (SEM). The SLNs were observed using a SEM (Tescan, Czech Republic) under specific conditions. The images were captured at an appropriate magnification to assess the size, shape, and uniformity of the nanoparticles.

###  In Vitro Drug Release

 The release profile of doxorubicin from the optimized SLN formulations was determined using the dialysis bag method.^20^ Cellulose membrane dialysis tubing (MWCO 10 kDa) was used to separate the doxorubicin from the nanoparticles. The nanoparticles, containing 5 mg of doxorubicin, were dispersed in 10 mL of distilled water and placed inside the dialysis bag.

 The dialysis bag was then immersed in 100 mL of phosphate-buffered saline (PBS, pH 7.4). The release was carried out in a shaking incubator at 37°C and 120 rpm. At predetermined time intervals (0.25, 0.5, 1, 2, 3, 4, 6, 8, 12, 24, 48, and 72 hours), 1 mL of the surrounding buffer was withdrawn and replaced with 1 mL of fresh pre-warmed PBS to maintain a constant volume. The released drug concentration was measured using UV-visible spectrophotometry at a wavelength of 480 nm. The UV spectrophotometry method (at 480 nm) was validated per ICH guidelines, showing linearity (R^2^ = 0.992 over 0.1–50 µg/mL), limit of detection (LOD = 0.05 µg/mL), limit of quantification (LOQ = 0.15 µg/mL), precision (intra-day CV < 5%), and accuracy (recovery 98–102%) using spiked PBS samples.

 All measurements were performed in triplicate, and the cumulative release percentage of doxorubicin was calculated.

###  Diabody Synthesis

 Gene Cloning and Transformation: The dual-functional diabody, targeting both CD44 and EGFR, was synthesized by cloning the respective genes into the expression vector pET-28. The recombinant plasmid was then transformed into *Escherichia coli* TOP10 for amplification of the gene copies. The plasmid was subsequently transferred into *E. coli* BL21 for protein expression.

 Step 1 (Expression): The expression of the diabody was induced in E. coli BL21 cells by adding IPTG (isopropyl β-D-1-thiogalactopyranoside) to the bacterial culture to initiate protein production.

 Step 2 (Purification): The diabody was purified using Ni-NTA affinity chromatography (Sigma-Aldrich, USA), following the manufacturer’s instructions to selectively bind the recombinant protein to the nickel resin.

 Step 3 (Purity Confirmation): The purity of the purified diabody was confirmed by SDS-PAGE and Coomassie Brilliant Blue staining to ensure that the diabody was free from contaminants also, Endotoxin levels in the recombinant diabody were measured via Limulus Amebocyte Lysate (LAL) assay (Pierce kit), confirming < 0.1 EU/mg to minimize bias in cell responses.

###  Conjugation of Diabody to SLNs

 The purified diabody was conjugated to the surface of solid lipid nanoparticles (SLNs) using a carbodiimide cross-linking method. The process involved the activation of the carboxyl groups of the diabody with EDC and NHS, followed by coupling the activated diabody to the lipid nanoparticles.^21^ Random multi-point attachment is possible; future optimizations could use site-specific mutants.

 Step 1: The activated diabody was mixed with the prepared SLNs in a reaction buffer, allowing for the formation of the conjugate.

 Step 2: The reaction mixture was incubated for 4 hours at room temperature to enable the formation of a stable conjugate between the diabody and the SLNs.

 Step 3: Unreacted reagents were removed by dialysis using a dialysis membrane (MWCO 10 kDa) against PBS at 4°C overnight to purify the conjugated SLNs.

###  Cytotoxicity Evaluation

####  MTT Assay for Cytotoxicity

 The cytotoxicity of the doxorubicin-loaded SLNs, with and without diabody conjugation, was evaluated in TNBC cell lines (e.g., MDA-MB-468)^22,23^.

- Step 1: Cells were seeded in 96-well plates at a density of 5,000 cells per well and incubated for 24 hours in a 37°C incubator with 5% CO₂.
- Step 2: Cells were treated with different concentrations of doxorubicin-loaded SLNs (5, 10, 20, and 50 µg/mL) and free doxorubicin for 48 hours.
- Step 3: After the treatment period, MTT reagent (5 mg/mL) was added to each well and incubated for 4 hours.
- Step 4: The absorbance was measured at 570 nm using an ELISA plate reader (Biotek, Synergy HTX, Turkey).
- Step 5: The cell viability was calculated as the percentage of viable cells compared to the control group.

###  Statistical Analysis

 All experiments were performed in triplicate, and the results are expressed as mean ± standard deviation (SD).

 One-way ANOVA with Dunnett›s post-hoc test, p = 0.012 at 0.5 µM, replicates n = 3, η^2^ = 0.45.

## Results

###  Preparation and Characterization of Doxorubicin-loaded SLNs

 As summarized in Table 2, the preparation of 15 experimental formulations was chosen for optimization experiments. The data were analyzed manually to determine the effects of different variables on the particle size and encapsulation efficiency. The independent variables (factors) considered for optimization were the total weight ratio of lipids (stearic acid and glycerol monostearate) and volume of aqueous phase, while particle size and encapsulation efficiency (EE%) were the dependent variables (responses).

**Table 2:** Conditions of Diabody conjugation SLN

	**1**	**2**	**3**	**4**	**5**	**6**	**7**	**8**	**9**
Diabody Concentration (µg/ml)	100	50	25	100	50	25	100	50	25
NHS Concentration (mM)	20	20	20	10	10	10	5	5	5
EDC Concentration (mM)	10	10	10	5	5	5	2.5	2.5	2.5

 The formulations varied based on these two independent variables, and the results were manually analyzed to identify the best formulation. The particle size of the formulations ranged from 120.85 nm to 396.65 nm (Table 1).

Figure 1-A shows that nanoparticle size increases with lipid amount (e.g., from 120.85 nm at 10 mg lipid to 396.65 nm at 20 mg), but this is modulated by aqueous phase volume, with optimal sizes (116–200 nm) at 15–20 mL due to improved emulsification. For low lipid (10 mg), EE% increases with aqueous volume up to 20 mL (peaking at 84.42%), then plateaus.

**Figure 1:**
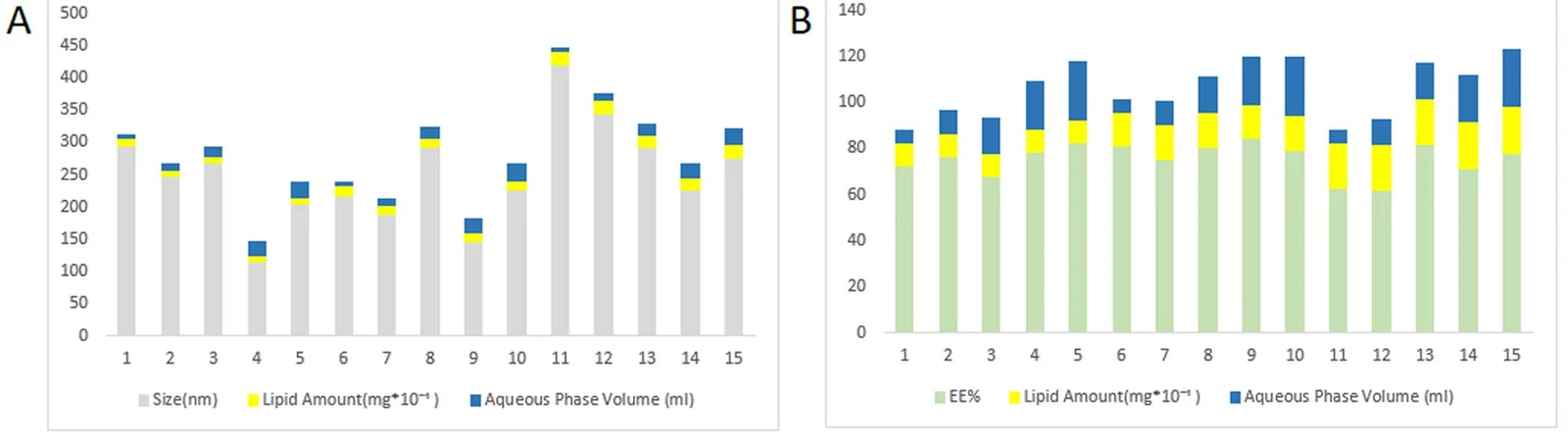
Effect of Lipid Amount and Aqueous Phase Volume on: A) Nanoparticle Size and B) Encapsulation Efficiency (EE%) respectively

 These results suggest that by controlling both lipid amount and aqueous phase volume, nanoparticle size can be systematically tailored. To produce smaller particles, reducing the lipid amount or increasing the aqueous phase volume (up to a certain limit) may be beneficial.

 As the aqueous phase volume increases, the EE% rises in formulations with a low lipid amount (10 mg), peaking at 82.55% with 25 mL. For moderate lipid amounts (15 mg), EE% increases and peaks at 84.42% at 20 mL, then decreases. In high lipid formulations (20 mg), EE% fluctuates, reaching a maximum of 81.84% at 15 mL.

 Overall, higher aqueous phase volumes generally improve EE%, but the lipid amount significantly influences this effect. The best balance between EE% and size was achieved with 10 mg lipid and 20 mL aqueous phase volume.

###  Evaluation of Optimized Doxorubicin-Loaded SLNs

 In this study, solid lipid nanoparticles (SLNs) loaded with doxorubicin were successfully prepared using a double-emulsion (w/o/w) method, followed by solvent evaporation and lyophilization.

 The resulting nanoparticles exhibited an average particle size of approximately 116.2 ± 10 nm (Figure 1), which is within the optimal range for drug delivery systems, ensuring efficient cellular uptake and sustained release of the drug. The PDI of the SLNs was found to be 0.25 ± 0.05, indicating a relatively narrow size distribution and high uniformity of the particles. Post-conjugation polydispersity index (PDI) was 0.32.

 The zeta potential of the SLNs was measured at -25.4 ± 3 mV, indicating good stability in aqueous suspension. Negative zeta potential values are typically associated with stable nanoparticle dispersions, as repulsive forces prevent particle aggregation.

 EE% of doxorubicin in the SLNs was calculated to be 78.78 ± 5%, demonstrating a high drug-loading capacity. This high EE suggests that the SLNs are efficient at encapsulating doxorubicin, minimizing drug leakage during circulation and enhancing the potential for targeted delivery.

 The morphology of the doxorubicin-loaded solid lipid nanoparticles (SLNs) was analyzed using Field Emission Scanning Electron Microscopy (SEM). As shown in Figure 2, the nanoparticles exhibited good dispersity, indicating effective encapsulation of the drug and minimal aggregation. These characteristics support the successful preparation of the SLNs for drug delivery applications.

**Figure 2:**
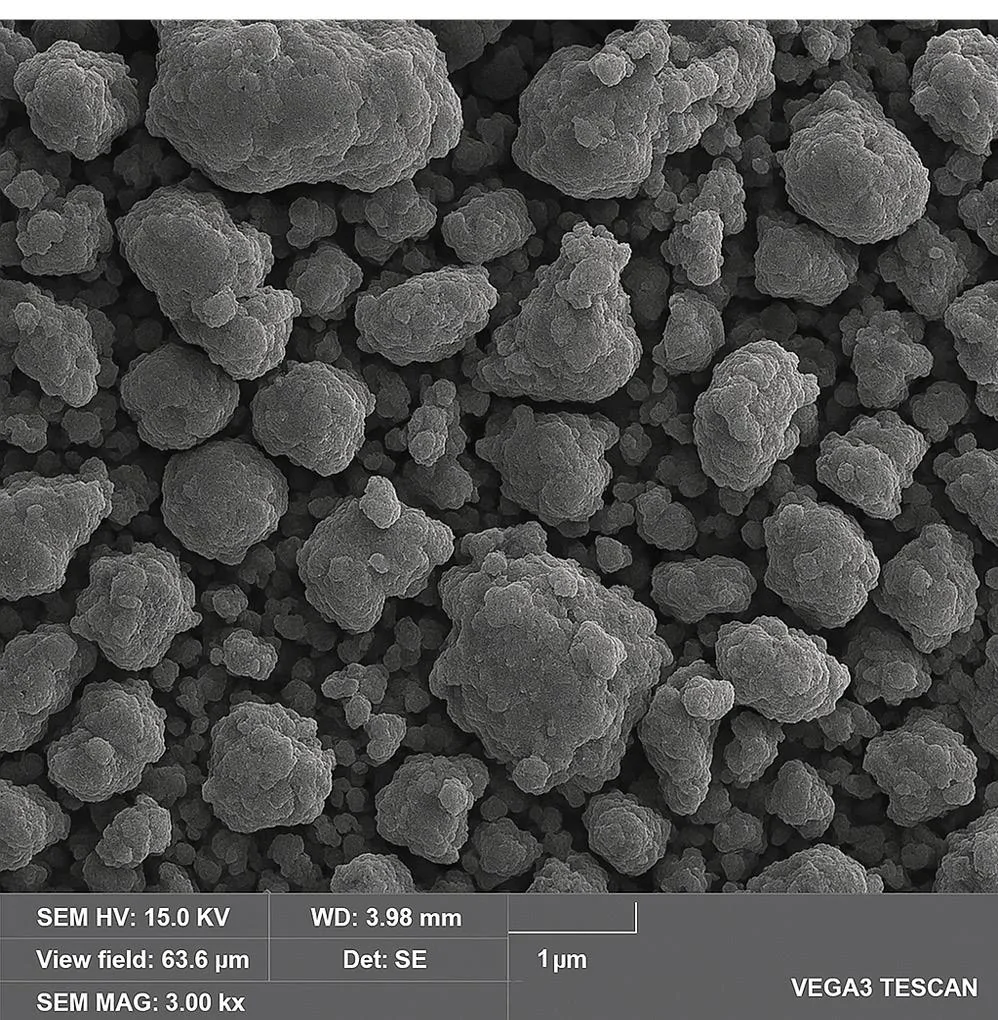
SEM images of doxorubicin-loaded SLNs

 The release of doxorubicin from the diabody-coated SLNs was studied in phosphate-buffered saline (PBS) at pH 7.4, simulating physiological conditions. The release profile showed a sustained release of doxorubicin over a period of 72 hours (Figure 3), with approximately 60% of the drug released after 48 hours and 80% after 72 hours. This controlled release profile suggests that the SLNs can provide a prolonged therapeutic effect while minimizing peak drug concentrations, thus reducing systemic toxicity. The release pattern follows a typical controlled release behavior, with an initial burst release followed by a sustained release phase, indicating the successful encapsulation and slow release of the drug from the nanoparticles.

**Figure 3:**
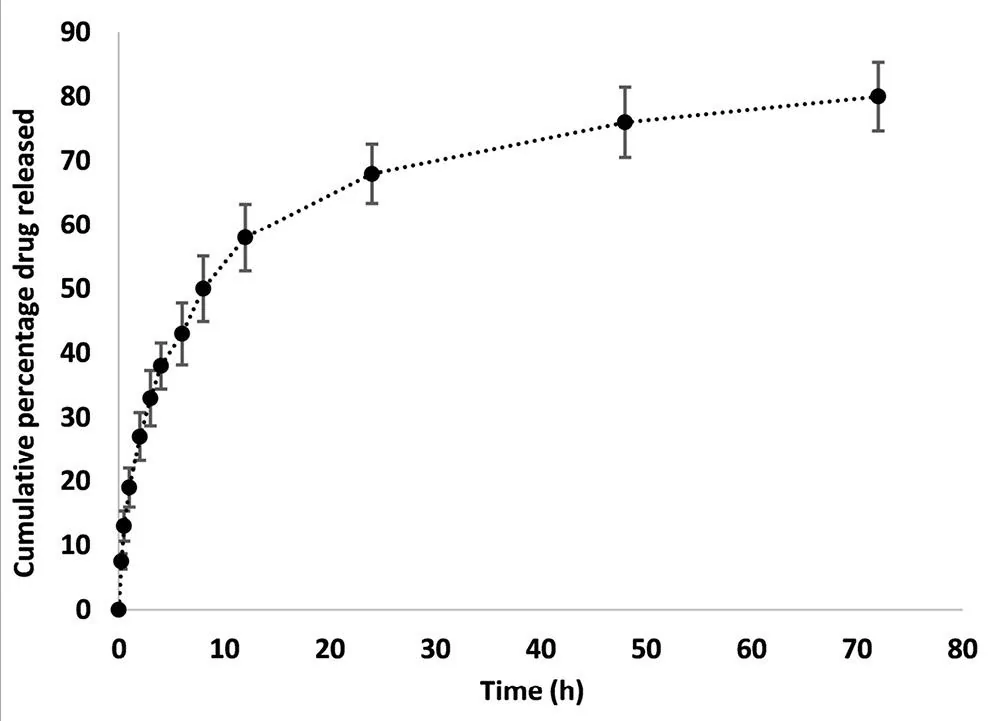
Cumulative Release Profile of Doxorubicin from SLNs

###  Synthesis and Purification of Diabody

 The dual-functional diabody, targeting both CD44 and EGFR, was synthesized through gene cloning and expression in Escherichia coli. The protein was subsequently purified using Ni-NTA affinity chromatography, which is a common method for purifying His-tagged recombinant proteins.

 As shown in Figure 4, the purification process was monitored using SDS-PAGE. The gel image demonstrates the successful purification of the diabody across different eluents. The SDS-PAGE gel shows clear bands corresponding to the diabody in the Elution 1, Elution 2, and Elution 3 fractions, confirming the presence of the recombinant protein. The final purified diabody was confirmed to be of the expected molecular weight, with a band observed around 53 kDa, consistent with the theoretical size of the diabody.

**Figure 4:**
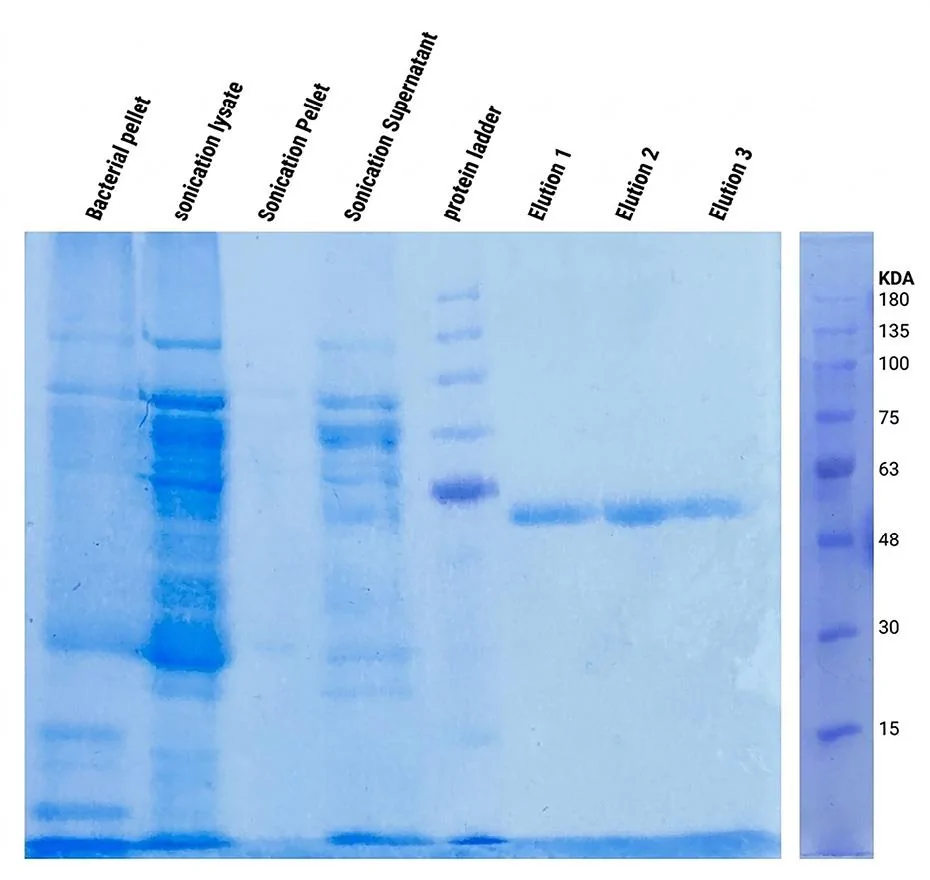
SDS-PAGE Analysis of Diabody Purification

 The protein was further analyzed for purity, and the results indicated a high degree of purity following the purification process, making it suitable for further conjugation to solid lipid nanoparticles (SLNs) for targeted drug delivery applications.

###  Covalent Conjugation of Diabody to SLNs

 In this study, diabody molecules were successfully conjugated onto the surface of SLNs through carbodiimide-mediated covalent bonding, employing EDC and NHS as coupling agents. To optimize the conjugation conditions, 9 different experimental conditions with varying concentrations of diabody, NHS, and EDC were tested (Table 2). The optimal condition for diabody conjugation was found to be: protein concentration of 100 µg/mL, NHS concentration of 5 mM, and EDC concentration of 2.5 mM. Under these conditions, the highest coupling efficiency achieved was approximately 49%, as calculated by measuring the free diabody concentration in the supernatant after the conjugation reaction at 280 nm (λmax of diabody). Post-conjugation, pelleted nanoparticles were analyzed via SDS-PAGE, revealing a distinct band at 53 kDa confirming covalent diabody attachment (updated Figure 4 with uncropped gel). Mass balance showed 95% protein recovery (bound + free).

 The coupling efficiency was calculated using the following equation:


$$Coupling\;Efficiency\;(\%)=\frac{Initial\;Diabody\;concentration-Final\;Diabody\;concentration}{Initial\;Diabody\;concentration}\times 100$$

To further validate the conjugation process and accurately quantify the amount of diabody attached to the SLNs, the Bradford protein assay was employed. In this method, the free protein concentration was determined by measuring absorbance at 595 nm, after reaction with Coomassie Brilliant Blue dye. The protein amount bound to nanoparticles was then calculated by subtracting the free protein concentration in the supernatant from the initial diabody concentration.

 The optimization results, as indicated in (Figure 5), demonstrate that decreasing the concentrations of EDC and NHS generally led to an increase in diabody coupling efficiency, while higher diabody concentrations showed a positive correlation with improved protein binding.

**Figure 5:**
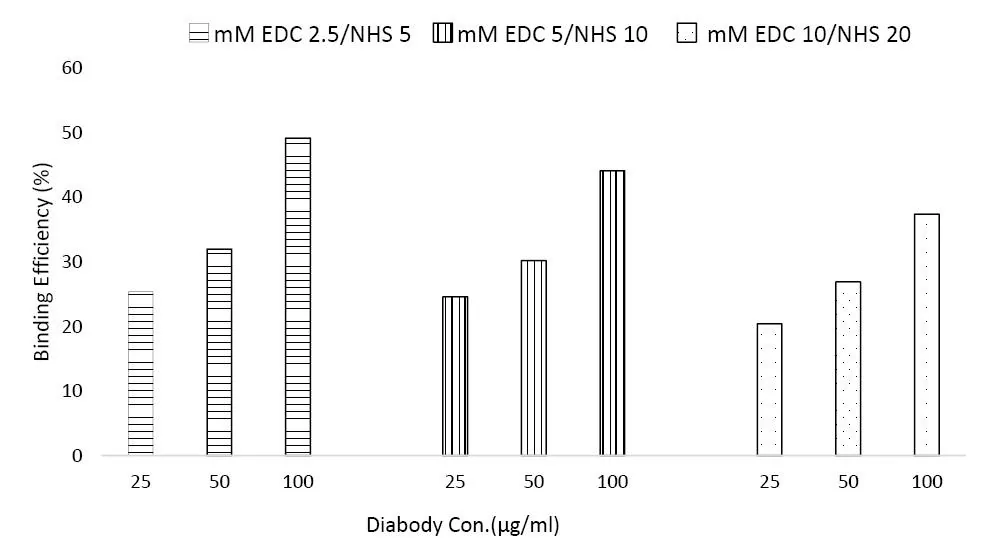
Binding efficiency of diabody conjugated to SLNs at different concentrations of diabody and varying EDC/NHS concentrations

 To confirm successful diabody conjugation and evaluate changes in nanoparticle characteristics, particle size and zeta potential were measured before and after diabody attachment (Figure 6).

**Figure 6:**
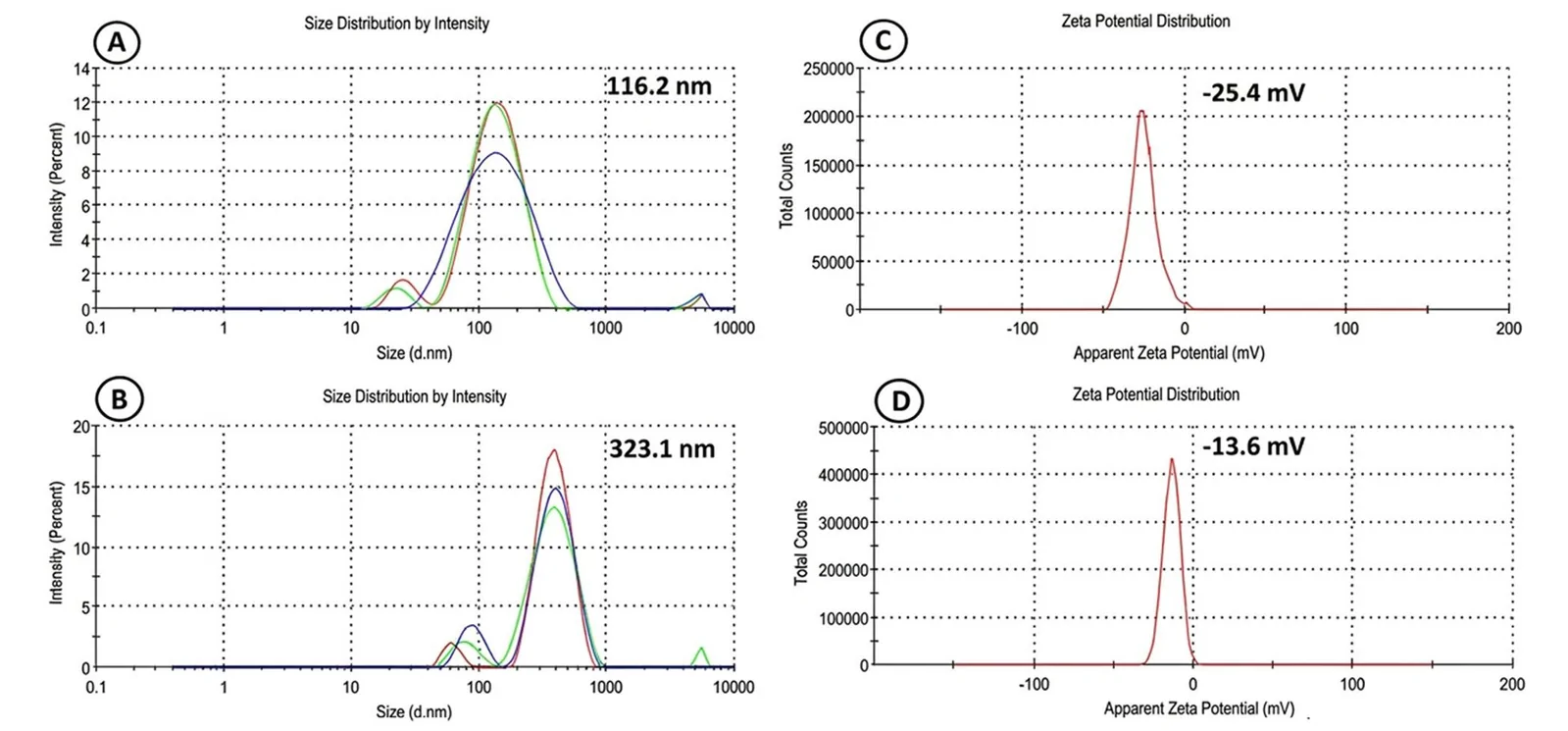
Particle charge size and nanoparticle size. Nanoparticle size A) before binding to diabody B) after binding to diabody; zeta potential of nanoparticle C) before binding to diabody, D) after binding to diabody

- Prior to diabody conjugation, SLNs exhibited an average particle size of approximately (116.2 nm) and a zeta potential of (-25.4 mV).
- Following diabody conjugation, the particle size increased to (323.1 nm), confirming successful diabody attachment on the nanoparticle surface. Additionally, the zeta potential shifted to (-13.6 mV), indicating significant changes in surface charge distribution due to diabody binding.

These alterations in nanoparticle characteristics (both size and zeta potential) further validated the effective covalently-bound conjugation of diabody molecules onto the SLN surface.

###  In Vitro Cytotoxicity Evaluation

 To evaluate the cytotoxic efficacy and targeting ability of the prepared nanoparticles, an MTT assay was performed using a triple-negative breast cancer cell line (MDA-MB-468). Cells were exposed to various concentrations of free doxorubicin (Dox), Dox-loaded SLNs (Dox-SLN), and diabody-conjugated Dox-SLNs (Dox-SLN-Diabody) for 48 hours, and cell viability was subsequently determined.

 The results (Figure 7) clearly indicate a concentration-dependent cytotoxic effect of all tested formulations. At lower concentrations (0.05 and 0.1 µM), no significant differences in cell viability were observed between free Dox, Dox-SLN, and Dox-SLN-Diabody groups. However, as the concentration increased (0.5 and 1 µM), notable differences emerged among the treatment groups.

**Figure 7:**
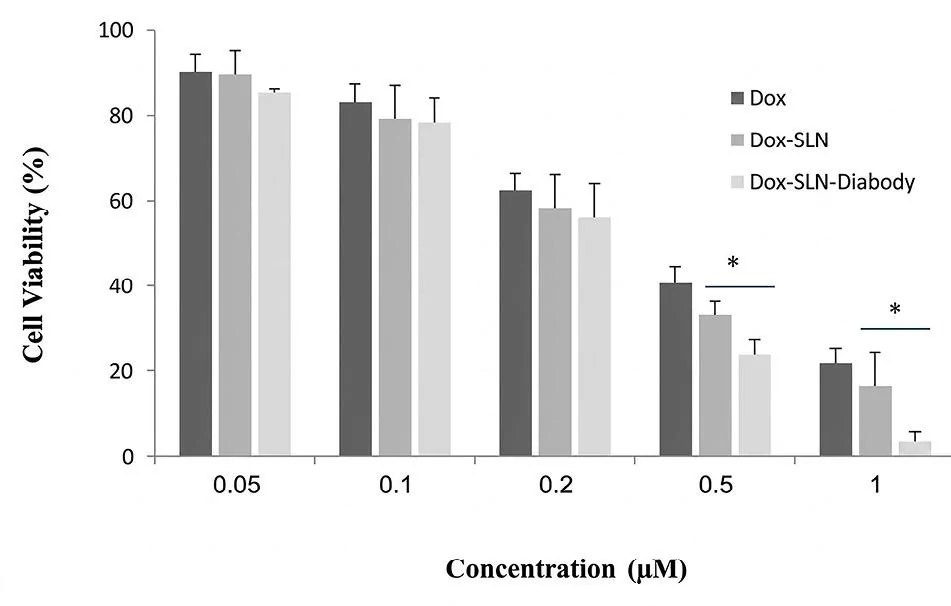
Cell viability comparison at various concentrations of doxorubicin, doxorubicin-loaded SLNs, and doxorubicin-loaded SLNs conjugated with diabody

 At concentrations of 0.5 µM and 1 µM, diabody-conjugated SLNs (Dox-SLN-Diabody) exhibited significantly higher cytotoxicity compared to free Dox and non-conjugated Dox-SLNs (*p < 0.05). Specifically, at the highest concentration (1 µM), Dox-SLN-Diabody treatment resulted in the lowest cell viability (~10%), clearly demonstrating enhanced targeted delivery and cellular uptake facilitated by the dual-functional diabody. MTT blanks with doxorubicin (no cells) showed < 5% absorbance interference at 570 nm.

 Overall, these findings indicate that conjugation of diabody molecules to the SLNs significantly enhances the therapeutic efficacy of doxorubicin against triple-negative breast cancer cells, likely due to improved cellular internalization mediated by targeted binding to specific receptors (EGFR and CD44) on the cell surface.

## Discussion

###  Interpretation of Results

 In this study, doxorubicin-loaded solid lipid nanoparticles (SLNs) were successfully formulated using the double-emulsion method. The particle sizes ranged between 116.2–345.18 nm, with a PDI of 0.25 ± 0.05, indicating good uniformity and suitability for drug delivery. Zeta potential was -25.4 ± 3 mV, suggesting colloidal stability. The high encapsulation efficiency (78.78 ± 5%) demonstrates the SLNs’ capacity to retain the drug and minimize leakage during circulation. Higher EE% values were observed with increasing aqueous phase volume, particularly at lower lipid concentrations—consistent with prior findings such as Ehrler et al. (2007).^21^

 The in vitro drug release showed a sustained pattern, with ~60% release after 48 hours and ~80% after 72 hours. This controlled release supports prolonged therapeutic activity and reduced dosing frequency. The release profile followed Higuchi kinetics (R^2^ = 0.95), with sink conditions confirmed (doxorubicin solubility > 10x released amount). Membrane controls (free doxorubicin) showed 95% diffusion in 24 h.

 Covalent conjugation of diabody to SLNs using EDC/NHS chemistry achieved a maximum coupling efficiency of 49% at optimal concentrations (100 µg/mL diabody, 5 mM NHS, 2.5 mM EDC). This conjugation increased nanoparticle size to 323.1 nm and shifted zeta potential to -13.6 mV, confirming successful attachment and surface modification.

 MTT assays on MDA-MB-468 cells showed that Dox-SLN-Diabody had the greatest cytotoxicity at higher concentrations (0.5–1 µM), reducing viability to ~10% at 1 µM. These results emphasize the enhanced cellular uptake and targeting efficacy of the diabody-conjugated formulation, supporting targeted therapy strategies.

###  Comparison with Previous Studies

 The results align with studies like Siddhartha et al. (2018)^24^, where bioconjugated nanoparticles showed enhanced uptake and efficacy in cancer models. Similarly, Ehrler et al. (2007) reported improved delivery via ligand-functionalized SLNs.

###  Strengths and Innovations

 This work demonstrates an efficient method for diabody conjugation onto SLNs, improving both drug delivery precision and cytotoxic efficacy. The approach integrates targeted delivery with lipid nanoparticle platforms in a TNBC model, representing a potentially translational cancer therapy strategy.

## Limitations and Challenges

 The cytotoxicity was only assessed in one cell line (MDA-MB-468). Broader testing across other cancer models and in vivo validation are needed. Moreover, the long-term stability of diabody-conjugated SLNs under physiological conditions remains unassessed and warrants further investigation.{Björgvinsdóttir, 2025 #2879}

## Future Directions

 Future studies should explore in vivo biodistribution, pharmacokinetics, and combination therapies using diabody-functionalized SLNs. Addressing manufacturing scalability and regulatory compatibility will also be crucial for clinical advancement.

 This study used one TNBC line (MDA-MB-468); future work should include non-TNBC comparators (e.g., MCF-7) and blocking/competition assays with excess anti-CD44/EGFR to confirm specificity. Retained diabody binding was not directly assessed (e.g., via ELISA or flow cytometry); serum stability and in vivo validation are recommended.

## Conclusion

 In this study, we have successfully developed doxorubicin-loaded solid lipid nanoparticles (SLNs) conjugated with a dual-functional diabody, targeting CD44 and EGFR, for the treatment of triple-negative breast cancer (TNBC). The optimized SLNs exhibited favorable characteristics, including a controlled drug release profile, uniform morphology, high encapsulation efficiency, and stable dispersion in aqueous solutions. The conjugation of the diabody to the SLNs significantly enhanced their targeting specificity and cytotoxic efficacy in vitro, especially against TNBC cells, as demonstrated by the MTT assay results.

 The diabody-conjugated SLNs showed a concentration-dependent increase in cytotoxicity at higher concentrations, with nearly 90% cell death at the highest tested concentration, compared to free doxorubicin or non-conjugated SLNs. These results strongly suggest that the diabody conjugation significantly improves the therapeutic efficacy of doxorubicin by facilitating its targeted delivery and enhanced cellular uptake in cancer cells.

 This study highlights the potential of diabody-conjugated SLNs as a promising nano carrier system for targeted drug delivery in cancer therapy, particularly for triple-negative breast cancer, where existing treatment options are often limited. However, further in vivo studies and clinical trials are needed to fully validate the long-term stability, pharmacokinetics, and therapeutic outcomes of these targeted formulations.

 In conclusion, this work provides valuable insights into the development of targeted nano medicine and paves the way for the clinical translation of diabody-conjugated lipid nanoparticles as an effective treatment strategy for cancer therapies.

## Competing Interests

 The authors declare no conflict of interest regarding the publication of this manuscript.

## Consent for Publication

 The authors consent to the publication of this manuscript in Advanced Pharmaceutical Bulletin.

## Data Availability

 The datasets generated and/or analyzed during the current study are available from the corresponding author on reasonable request.

## Ethical Approval

 This study was aproved by Hamadan University of Medical Scieneces (Ethical code: IR.UMSHA.REC.1399.717).
